# Transitioning from native to synthetic receptors: broadening T-cell engineering and beyond

**DOI:** 10.1038/s41423-025-01304-8

**Published:** 2025-06-06

**Authors:** Li Yu, Yue Liu, Xin Lin

**Affiliations:** 1 Changping Laboratory, Beijing, China; 2 School of Tsinghua Medicine, Tsinghua University, Beijing, China

**Keywords:** T-cell receptor, CAR, TCR-like receptors, Immunotherapy, Tumour immunology, Cancer immunotherapy

## Abstract

T-cell immunotherapy has progressed rapidly, evolving from native T-cell receptor biology to the development of innovative synthetic receptors that extend therapeutic applications beyond cancer. This review explores engineering strategies, ranging from natural TCRs to synthetic receptors, that increase T-cell activation and therapeutic potential. We begin by highlighting the foundational role of native receptors in the T-cell response, emphasizing how these structural and functional insights inform the design of next-generation synthetic receptors. Comparisons between CAR and TCR-like synthetic receptors underscore their respective advantages in specificity, efficacy, and safety, as well as potential areas for further improvement. In addition, gene editing technologies such as CRISPR-Cas9 enable precise modifications to the T-cell genome, enhancing receptor performance and minimizing immunogenic risks. In addition to tumors, these engineered T cells can be directed against viral infections, autoimmune disorders, and other diseases. We also explore advanced strategies that engage multiple immune cell types to achieve synergistic, durable responses. By demonstrating how native and synthetic receptors collectively drive innovation, this review aims to inspire new research directions and ultimately expand the scope of T-cell engineering for universal therapeutic applications.

## Introduction

T cells play a pivotal role in defending against infection and cancer. The highly specific recognition of pathogens and tumor cells is mediated by the T-cell receptor (TCR), which undergoes somatic rearrangement and mutation to achieve vast antigen diversity. Upon engaging with extracellular antigens, TCRs initiate intracellular signaling cascades that drive T-cell proliferation and effector function, guided by the nature of TCR signaling and the T-cell subset involved. T-cell activation is a coordinated process involving numerous membrane and intracellular proteins [1]. The primer trigger is the ligation of the peptide/MHC (pMHC) complex to the TCR, which is then modulated and strengthened by coreceptors, adhesion molecules and signaling proteins and nonprotein cofactors, eventually leading to transcriptional and epigenetic reprogramming.

Inspired by T-cell activation, the earliest chimeric antigen receptor (CAR) incorporated extracellular domains from immune receptors (CD4, CD8 or CD25) with signal transduction provided by the cytoplasmic tail of the ζ chain, containing three ITAMs sufficient for T-cell activation [2–4]. First-, second- and third-generation CARs replace the extracellular domain with a single-chain variable fragment (scFv) specific to tumor antigens, while their cytoplasmic domain comprises the ζ chain and one (second generation) or two (third generation) costimulatory receptors, such as 4-1BB or CD28 [5–9]. CAR-T-cell therapy has achieved remarkable success in treating hematopoietic tumors, particularly second-generation CARs, which are twelve CAR-T-cell products approved by the FDA. However, CAR-T-cell therapy shows limited efficacy in solid tumors, possibly due to early T-cell exhaustion prior to tumor infiltration. These findings indicate the need for further CAR optimization to meet the requirements of treating solid tumors [10].

In this review, we first introduce the mechanism by which native receptors drive T-cell activation and illustrate how these principles guide the design and modification of CARs and other novel TCR-based synthetic receptors. We aim to synthesize current insights into native T-cell activation to inform the rational development of synthetic receptors and improve their therapeutic performance. After reviewing the fundamental aspects of native T-cell activation, we discuss how these functional motifs are incorporated into CARs and other innovative synthetic receptors. Finally, we offer broader perspectives that extend beyond T-cell engineering, aiming to foster more universal applications of immunotherapies.

## Native receptors: key drivers of T-cell activation and response

**Fig. 1:**
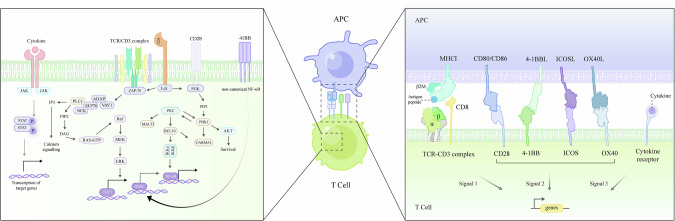
Native receptors contributing to T-cell activation and response. Key native receptors involved in T-cell activation, including the TCR–CD3 complex, costimulatory receptors, and cytokine receptors, cooperate to initiate and regulate T-cell activation, proliferation, and differentiation. The classic three-signal model is depicted as follows: (1) antigen-specific recognition via the TCR–CD3 complex (assisted by CD4/CD8 binding to pMHC), (2) costimulatory receptor engagement (e.g., CD28, ICOS, 4-1BB, OX40), and (3) cytokine-mediated signals that guide T-cell differentiation and survival. Together, these receptors orchestrate robust immune responses against infections and cancers

T cells depend on multiple native receptors, including the TCR-CD3 complex, costimulatory/inhibitor receptors, cytokine receptors and chemokine receptors, to recognize antigens and initiate the immune response (Fig. 1). Together, these native receptors orchestrate T-cell activation, proliferation and differentiation, enabling robust defense against infection and cancer.

According to the three-signal model of T-cell activation (Fig. 1), signal 1 is provided by the TCR-CD3 complex upon binding to its cognate peptide-MHC (pMHC) complex [11]. CD8 or CD4 molecules further assist this signal by interacting with MHC class I or class II molecules, respectively [12]. Signal 2 arises from costimulatory receptors, such as immunoglobulin superfamily members (e.g., CD28 and ICOS) and tumor necrosis factor receptor superfamily members (e.g., 4-1BB and OX40), which are essential for completing T-cell activation and enhancing effector function, survival and expansion [13]. Signal 3 is provided by cytokines that facilitate T-cell differentiation and sustain viability [14]. Next, we briefly outline how these three signals collectively trigger T-cell activation and govern immune responses.

### TCR-CD3 complex

Signal 1 from the TCR-CD3 complex confers antigen specificity and drives extensive intracellular signaling. Although TCR engagement with pMHCs is known to induce the phosphorylation of CD3 cytoplasmic tails (a process termed “TCR triggering”), the precise mechanism remains to be explored. Several models have been proposed to explain TCR triggering. In the aggregation model, ligand-induced clustering of the TCR-CD3 complex increases the proximity of associated Lck molecules to CD3, increasing CD3 phosphorylation [15, 16]. The segregation model supposes that pMHC binding excludes the inhibitory phosphatase CD45 from the TCR–CD3 complex, allowing CD3 phosphorylation to proceed [17]. Two additional models describe the TCR-CD3 complex as an allosterically regulated macromolecule. In the allosteric model, antigen binding induces conformational changes that expose previously concealed phosphorylation sites on the cytoplasmic domain of CD3 [18]. In the mechanosensing model, the mechanical force generated by interacting with T cells and target cells pulls the CD3ζ cytoplasmic tail away from the membrane, allowing Lck and other kinases to phosphorylate these exposed sites [19]. These mechanisms likely act in concert to accomplish TCR triggering. In addition to the molecular mechanism, TCR ligation initiates the formation of the immunological synapse (IS), which further amplifies TCR signaling and effector responses. During T-cell activation, TCR microclusters engage pMHCs and migrate centrally, forming a concentric ring-like structure. TCRs and pMHCs occupy the core and are surrounded by costimulatory molecules, kinases and adhesion proteins, whereas inhibitory receptors segregate to the periphery [20, 21]. This arrangement increases signal strength and enables T cells to distinguish themselves from nonself cells with high specificity.

Signal transduction by the TCR-CD3 complex is mediated through the immunoreceptor tyrosine-based activation motif (ITAM) on the cytoplasmic domain of the CD3 subunits. CD3γ, CD3δ and CD3ε each possess a single ITAM, whereas the CD3ζ chain has three, yielding a total of ten ITAM motifs per TCR‒CD3 complex. Each ITAM carries two tyrosine residues that, once phosphorylated, recruit signaling proteins containing the SH2 domain [22, 23]. With the assistance of the CD4 or CD8 coreceptor, the Src family kinase Lck, is recruited to phosphorylate these ITAM tyrosine residues. Phosphorylated ITAMs then recruit ZAP70, which is activated by Lck. Activated ZAP70 kinase subsequently phosphorylates the scaffold protein LAT and the adapter protein SLP-76, forming the LAT signaling complex. The complex coordinates four major downstream signaling pathways [23–25]. (1) Recruitment of ADAP to upregulate integrin expression for adhesion and migration. (2) Activation of Vav promotes actin polymerization and cytoskeletal remodeling. (3) Production of PIP3 by PI3K and activation of the AKT pathway. (4) Recruitment and activation of PLCγ1 lead to three key transcriptional programs via NFAT, NF-κB and AP-1 [26–28]. Collectively, these events drive robust gene expression, guiding T-cell effector function, differentiation, and long-term persistence.

### Costimulatory receptors

#### CD28

CD28 consists of two cytoplasmic motifs, YMNM and PYAP, which directly influence T-cell activation. The YMNM motif is associated with the p85 subunit of PI3K, triggering the PI3K-AKT pathway and promoting T-cell proliferation and survival [13]. Upon B7 binding, CD28 becomes tyrosine-phosphorylated (possibly by Lck and/or Fyn), facilitating PI3K recruitment and activation. Activated PI3K then produces PIP3 and other phospholipid mediators [29]. By lowering the threshold for TCR triggering and shortening the stimulation time required for T-cell commitment, CD28 signaling both amplifies and sustains the TCR response [30, 31]. CD28 engagement also promotes Vav1 hyperphosphorylation, which drives cytoskeletal remodeling and coalesces membrane rafts around the TCR signaling complex [32, 33]. These rafts contain key members of the Ras/MAPK and SAPK/JNK pathways that activate c-Fos and c-Jun, forming the AP-1 transcription factor. AP-1, in turn, promotes IL-2 expression and stabilizes its mRNA while also upregulating the antiapoptotic gene Bcl-xL [34]. Additionally, CD28 signaling augments NF-κB activation, supporting the production of cytokines (e.g., IL-2, IL-4, IL-6, IFN-γ, and MIP-1α), cytokine/chemokine receptors (e.g., IL-2R, IL-12R, and CXCR5) [33], and further costimulatory molecules (CD40L, ICOS, OX40, and 4-1BB) [35]. Genome-wide analysis indicated that CD28 primarily increases TCR-triggered gene expression rather than inducing a unique set of genes and drives these genes to even higher expression levels [35, 36]. By inhibiting GSK3, CD28 prevents NFAT export from the nucleus and prolongs NFAT-mediated transcription [37].

CD28 is crucial for both naïve and memory T-cell activation, with naïve T cells particularly reliant on CD28 costimulation alongside TCR signals for full activation and clonal expansion [38, 39]. In addition to its role in early activation, CD28 also modulates the metabolism of PD-1-expressing, stem-like CD8 T cells, promoting self-renewal and differentiation. By reinforcing TCR signaling, CD28 stimulates pathways such as the PI3K–AKT–mTOR pathway, which enhances cytokine production and cellular proliferation. Increasing CD28 costimulation in exhausted T cells can thus shift stem-like populations toward more robust, effector-like states, helping to overcome inhibitory signaling in chronic infection or tumor microenvironments [40].

#### 4-1BB

4-1BB (also known as CD137) is a member of the tumor necrosis factor receptor superfamily (TNFRSF), which also includes OX40, CD27, CD30, DR3, GITR and HVEM [13]. Unlike CD28, 4-1BB is absent on naïve T cells and is induced following T-cell activation. Once expressed, three monomers on the membrane form a trimeric complex and engage ligands on antigen-presenting cells (APCs). The cytoplasmic domains then recruit TNF receptor-associated factor (TRAF), which drives canonical and noncanonical NF‑κB signaling and the JNK, p38 MAPK, AP1, ERK and NFAT pathways [41, 42]. Depending on the T-cell activation and differentiation stage, 4-1BB cooperates with the TCR-CD3 complex to provide costimulatory signals, augmenting IL-2 production, proliferation and differentiation.

4-1BB also enhances T-cell survival by promoting antiapoptotic factors (BCL‑2, BCL‑XL, and BFL1) and activating AKT, which regulates cyclins and cyclin-dependent kinases [42, 43]. Critically, 4-1BB supports memory T-cell expansion and survival upon secondary challenge [44]. In some cases, 4-1BB stimulation in memory T cells is independent of TCR engagement. Treatment with 4-1BB agonists leads to robust proliferation without affecting naïve T cells or requiring cytokines such as IL-15 or IFN-γ [45]. The strong costimulatory function correlates with rapid, high-level expression on effector CD8^+^ T cells, increasing sensitivity to low-avidity TCR signals and promoting bystander activation [46–48]. Moreover, the administration of 4-1BB monoclonal antibodies enhances antitumor responses as effectively in CD4^+^ or NK cell-deficient mice as in wild-type mice, emphasizing the important role of CD8^+^ T cells [49]. Although Treg cells also rely on costimulatory signals, studies suggest that while 4-1BB may promote Treg generation and expansion, it can diminish their suppressive capacity [50–53].

#### Cytokine receptors

The cytokines are critical for the T-cell response include interleukins, interferons, the TNF superfamily, and chemokines [54–56]. These molecules are often grouped into superfamilies that share receptors or exhibit overlapping functions [57, 58]. For example, the IL-6 family (IL-6, IL-11, IL-27, ciliary neurotrophic factor (CNTF), leukemia inhibitory factor (LIF), oncostatin M (OSM), cardiotrophin 1 (CT-1) and cardiotrophin-like cytokine (CLC)) relies on at least one gp130 subunit and has both distinct and shared functions [59]. Similarly, type I interferons (IFN-α, IFN-β), type II interferons (IFN-γ) and type III interferons (IFN-λ) possess common immunoregulatory, antitumor and autoimmune properties [57]. Originally identified for its tumor necrosis effects, the TNF superfamily now encompasses 19 ligands and 29 corresponding receptors [54]. Chemokines are a group of small, structurally related molecules of approximately 8–14 kDa [60–62]. Currently, 47 chemokines that regulate cell trafficking and homeostasis through G protein-coupled receptors have been identified in humans [61].

Most cytokines initiate T-cell responses via receptor engagement, which activates the JAK/STAT pathway [63, 64]. Recently, Dr. Garcia’s group revealed a ligand-dependent dimerization process via cryo-electron microscopy [65]. The cytokine first binds a high-affinity subunit, which then recruits a low-affinity subunit, bringing together their intracellular Janus kinases (JAKs). JAKs transphosphorylate tyrosine residues in the cytoplasmic domains of the receptor, creating docking sites for signal transducer and activator of transcription (STAT) proteins, which, once phosphorylated, dimerize, translocate to the nucleus, and regulate gene expression [63]. Despite this mechanistic clarity, therapeutic applications remain challenging owing to the complexity of approximately 40 cytokines, 4 JAKs and 7 STATs, as well as the pleiotropic actions of cytokines that yield diverse functional responses across different cell types [14, 66].

## Synthetic receptors: leverage principles from native receptors

**Fig. 2:**
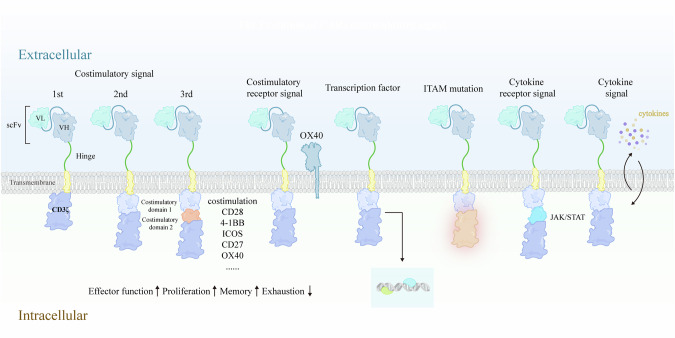
The evolution of CAR designs, inspired by native T-cell signaling. The first-generation CAR fused the single-chain variable fragment (scFv) to CD3ζ to provide signal 1. Second-generation CARs add costimulatory elements for signal 2, whereas third-generation constructs integrate two distinct costimulatory domains. Further modifications include costimulatory receptor signaling modules, transcription factors, ITAM mutations, and cytokine/cytokine receptor signals, which broaden CAR functionality, drawing from native T-cell receptor principles to improve activation and therapeutic efficacy

By the 1980s, the structural and signaling mechanisms of the TCR from ligand binding through cytoplasmic activation were largely elucidated, paving the way for the development of synthetic receptors such as chimeric antigen receptors (CARs). Compared with intricate TCRs, CARs are relatively simple single-chain constructs. Early CAR designs coupled the cytoplasmic tail of CD3ζ (with three ITAMs sufficient for T-cell activation) to CD4, CD8 or CD25 [2, 3]. Since then, subsequent modifications aimed to better mimic TCR activation and enhance therapeutic potency (Fig. 2).

### Structure of CAR and its evolution

CARs initially emerged by fusing an antigen-recognition domain from antibodies to a T-cell activation domain from the TCR [5, 67, 68]. Over time, this approach has evolved into a first-generation CAR, where two-chain antibody fragments are replaced by a scFv fused to CD3ζ [5]. Although first-generation CARs mediate MHC-independent T-cell recognition, they deliver only the TCR signal and lack a costimulatory signal, limiting their long-term antitumor efficacy [69, 70]. Recognizing the need for a second signal, Dr. Sadelain and colleagues demonstrated that overexpressing costimulatory receptors enhances T-cell proliferation and survival [71]. They further reported that fusing the CD28 signal domain into a first-generation CAR significantly strengthened the effector function and proliferation of CAR-T cells [7, 8]. The coexpression of CD80 and 4-1BBL subsequently increased antitumor efficacy [72], whereas Dr. Campana group enhanced in vivo expansion and therapeutic effects via the incorporation of 4-1BB signaling [73]. These two-signal constructs, which combine CD3ζ and one costimulatory domain (e.g., CD28, 4-1BB, ICOS, OX40 or CD27), are known as second-generation CARs [74] and are currently in widespread clinical use. Third-generation CAR-T cells are engineered for tumors with low antigen density, where signals for activation and survival are limited. The addition of two costimulatory domains is intended to compensate for this deficit. Many studies have reported greater toxicity and earlier T-cell exhaustion, likely caused by overstimulation from dual costimulatory signaling in third-generation CAR-T cells. Because existing data come from small, heterogeneous cohorts, they are insufficient to draw conclusions. Nevertheless, these findings underscore an important design principle: the functional effects of combining different signaling domains are neither purely additive nor linear, reflecting intricate T-cell-signaling networks [9, 75, 76].

### Substitution of the CD3ζ-ITAM-containing tails

Early studies underscored the importance of CD3 ITAMs and their phosphorylation dynamics for T-cell development and function [77, 78]. Because the TCRα/β heterodimer lacks inherent signal capacity, it pairs noncovalently with signal-transducing subunits (CD3γ, CD3δ, CD3ε and usually a ζ chain homodimer) [79]. Each CD3 chain contains a single ITAM, whereas each ζ chain has three tandem ITAMs, yielding up to 10 ITAMs in the αβTCR and γδTCR complexes [80, 81]. Despite their canonical YxxL/I-X_6-8_-YXXL/I motif, these ITAMs differ in amino acid composition and downstream effector binding, including interactions with ZAP-70, Shc, PI3K(p85), Grb2, Fyn, and Ras-GAP [77, 82, 83].

In second-generation CARs, the cytoplasmic tail of the CD3ζ chain (bearing three ITAMs) is commonly employed for T-cell activation [84]. Increasing the number of ITAMs can increase signaling efficiency, yet specific ITAM mutations can yield different effects: retaining the first ITAM while mutating the second and third ITAM (1XX) augments effector activity beyond that of the standard 1928ζ CAR, inducing sustained tumor regression in vivo. Mutating the first and second ITAMs (XX3) can produce highly persistent T cells [85]. Additionally, mutating the first and third ITAMs (X2X) reduces T-cell apoptosis and cytokine production [85]. In addition to the ζ chain, CD3ε, CD3γ and CD3δ also undergo phosphorylation upon TCR engagement [25], promoting efforts to engineer CARs containing these cytoplasmic domains [86]. These CARs outperform conventional ζ-based constructs in vivo [86], which is partly due to the phosphatase SHP1, which binds better to the phosphorylated C-terminal tyrosine of the CD3δ ITAM than to that of the CD3ζ ITAM and can fine-tune ligand discrimination thresholds [87, 88]. Moreover, a receptor kinase (RK)-binding motif in CD3ε recruits endogenous Lck upon TCR ligation, stabilizing activation signals. Incorporating this motif into a CD3ζ-based CAR (εRKζCAR) enhances antitumor efficacy both in vitro and in vivo by mimicking TCR-derived signals [89, 90]. Recently, partial deletions or specialized modifications to CD3ε have improved CAR surface expression while retaining cytotoxic function [91]. Dr. Xu’s group reported that an engineered CAR with a uniquely designed CD3ε module, E_B6I_, induces liquid–liquid phase separation to form stable immunological synapses, reducing receptor endocytosis and trogocytosis [92]. This design preserves antigen sensitivity, strengthens cytotoxicity, and enhances CAR-T-cell persistence against various tumors.

### Modification of the costimulatory domain

In first-generation CAR-T cells, continuous antigen stimulation often leads to T-cell exhaustion and limited antitumor efficacy. The incorporation of costimulatory signaling domains, which characterize second- and third-generation CARs, increases proliferation, persistence, cytotoxicity and memory differentiation. One study demonstrated that 4-1BB costimulation relies on K63-linked polyubiquitin to increase T-cell persistence and antitumor efficacy [93]. Receptor downmodulation and endosomal trafficking, which are essential for T-cell activation, also help avoid overstimulation. Adjusting these posttranslational processes can improve CAR-T-cell metabolism, expansion, and memory formation [93]. Alternatively, introducing a full-length costimulatory receptor into CAR constructs enables costimulation independent of tumor antigen recognition [94]. A screening of 12 costimulatory receptors revealed that combining CARs with OX40 was most effective. OX40 signaling diminished apoptosis by upregulating antiapoptotic Bcl-2 family genes and increased proliferation through activation of the NF-κB, MAPK, and PI3K–AKT pathways. In a phase I clinical trial for metastatic lymphoma, these OX40-expressing CAR-T cells persisted and reduced the tumor burden [94].

In addition to the integration of natural costimulatory domains, researchers have developed chimeric costimulatory receptors (CCRs). By replacing a scFv with the extracellular region of a costimulatory molecule, additional signaling is provided to support CAR-T-cell function. For example, combining a BCMA or CD19-CAR with a CD38-directed CCR increases tumor killing, even against cells expressing very low levels of the target antigen [95]. The CAR with CCR outperforms the traditional CD28ζ or 4-1BB ζ CAR. Pairing GD2/B7-H3 or MSLN/CSPG4 with CD28ζ/4-1BB signals confers rapid and prolonged antitumor effects as well as improved T-cell fitness [96]. Another variant, the chimeric switch receptor (CSR), converts an inhibitory checkpoint signal into a costimulatory signal. A broad screen revealed that TGFβR2-41BB is particularly effective at promoting T-cell function and tumor clearance [97], an approach that also improves CAR-T-cell therapy [98]. Moreover, PD1-CD28 competes with endogenous PD1 signaling and transform PDL1 engagement into an activating signal, enhancing central memory phenotypes and the Th1-like response [99–101]. Cotransducing a suboptimally activating ζCAR alongside a CCR ensures that both tumor antigens are recognized for full activation, limiting off-tumor toxicity and transmitting costimulatory signals [102]. When PSMA and PSCA are used as dual targets in prostate tumors, only double-positive tumors are eliminated, sparing single-positive tissues [102].

Recently, researchers have attempted to replace traditional CD3ζ domains on CARs with intracellular proximal T-cell signaling molecules, such as ZAP-70 CAR, which can activate T cells and eradicate tumors while bypassing upstream signaling proteins [103]. Alternatively, leveraging the cooperative role of LAT and SLP-76 to engineer a logic-gated intracellular network (LINK) CAR integrates signals in a Boolean-AND-gated CAR to increase efficacy and minimize on-target, off-tumor toxicity [103]. Overexpressing signaling downstream components, such as c-Jun, prolongs CAR-T-cell persistence, reduces exhaustion and improves antitumor potency [104], an approach under clinical evaluation in acute myeloid leukemia (AML) (NCT04835519) [105]. In pediatric neuroblastoma, engineered GPC2-CAR-T cells with c-Jun overexpression enhance tumor clearance even at low antigen density while mitigating off-target toxicity [106].

### Equipped CAR with cytokine signaling

Because cytokines can deliver a “third signal” to maintain the CAR-T-cell response, recent efforts have focused on incorporating natural or synthetic cytokine signaling, collectively referred to as fourth-generation, TRUCK or “armored” CAR-T cells [107–110]. For example, TRUCK-T cells can secrete one or more cytokines that not only enhance T-cell function but also remodel the tumor microenvironment (TME).

Improving T-cell persistence and proliferation is vital for effective CAR-T-cell therapy against solid tumors [111]. Several studies have armed CARs with autocrine cytokines such as IL-15, which can maintain CD8^+^ T cells, increase OXPHOS and promote memory formation, together with significantly upregulated Fos and Jun subfamily members [112–115]. In a phase I clinical trial, glypican-3 (GPC3) CARs expressing IL-15 achieved greater peak cell expansion and promising antitumor efficacy (50% partial response and 50% progressive disease). However, this effect is correlated with more severe cytokine release syndrome (CRS), which is manageable via IL-1/IL-6 blockade or an inducible caspase-9 safety switch (NCT04377932, NCT05103631) [116]. Similarly, incorporating IL-15 into GD2 CAR-T cells for retinoblastoma treatment has shown potential, with clinical trials ongoing for neuroblastoma and osteosarcoma (NCT03721068) [117]. Coexpressing IL-15 and IL-21 in GPC3 CAR further increased T-cell viability and antitumor ability in a hepatocellular carcinoma model (NCT02932956, NCT02905188) [118]. Although IL-2 promotes T-cell activation and expansion, systemic administration can cause serious adverse events [119]. Therefore, synthetic gene circuits (e.g., synNotch or synZiFTRs) have been employed to conditionally release IL-2, increasing T-cell infiltration and proliferation while reducing toxicity and Tregexpansion [119, 120]. Among the different cytokines that are constitutively expressed in CD19 CAR-T cells, IL-7 and IL-21 have the strongest antitumor effects, whereas IL-15 and IL-21 enhance long-term T-cell persistence [121]. Additionally, incorporating the IL-23 subunit IL-12β p40 can increase IL-23R expression on CAR-T cells, driving their proliferation and survival [122]. Some cytokines, such as IL-10, reprogram T-cell metabolism to counter T-cell exhaustion [123]. IL-10–Fc fusion proteins increase oxidative phosphorylation in exhausted CD8^+^ TILs [124], and CAR-T cells coexpressing IL-10 can eradicate solid tumors across multiple cancer types while also generating stem-like memory T cells [125].

Despite these benefits, constitutive cytokine expression may cause severe adverse events (e.g., neurotoxicity and CRS). One of the approaches to avoid this potential risk is to engineer the cytokine receptor itself. Coexpression with IL-7R in CAR-T cells sustains signaling upon antigen recognition while sparing bystander lymphocytes, with early clinical trials (NCT04099797, NCT03635632) evaluating IL-7R-expressing GD2 CAR-T cells in brain tumors and refractory neuroblastoma [126]. Since γC family cytokines (IL-2, IL-7, IL-15, IL-21, and IL-23) signal through the JAK-STAT pathway [63], researchers have also equipped CARs that encode the JAK-STAT motif directly. The incorporation of a truncated IL-2 receptor β-chain (IL-2Rβ) and a STAT3-binding YXXQ motif into a CD28ζ CAR (28-ΔIL2RB-z(YXXQ)) results in antigen-dependent JAK/STAT signaling, enhancing T-cell proliferation and preventing terminal differentiation [127]. These CAR-T cells outperform those with only the CD28 or 4-1BB costimulatory domains in both hematopoietic and solid tumor models [127]. Another approach is to limit the bioactive spectrum of cytokines to control their toxicity. Neo-2/15, a synthetic two-component mimetic of IL-2 and IL-15, remains inactive alone until its fragments colocalize with specific surface markers on tumor cells [128]. Under “transactivation”, Neo2/15 has antitumor activity with reduced systemic toxicity in melanoma models. In “*cis*-activation”, Neo2/15 selectively expands CD8^+^ T cells and promotes CAR-T-cell activity in a lymphoma xenograft model [128]. Similar receptor modifications also help minimize toxicity. Orthogonal IL-2/IL-2Rβs exclusively bind to each other [129], enabling IL-2 signaling to act only on CAR-T cells, avoiding the side effects of IL-2 on other immune cells [130, 131]. A first-in-human phase I trial (NCT05665062) evaluating CD19 CAR-T cells coexpressing orthogonal IL-2/IL-2Rβ in hematopoietic malignancies reported encouraging initial outcomes. Extending this concept, Dr. Garcia group designed chimeric receptors featuring an orthogonal IL-2Rβ extracellular domain fused to the intracellular domain from various γ_c_ receptors (e.g., IL-4, IL-7, IL-9 and IL-21) [132]. Among these receptors, the IL-2Rβ-ECD–IL-9R-ICD construct activates STAT1, STAT3, and STAT5, endowing T cells with both stem cell memory and effector characteristics. This approach outperforms orthogonal IL-2/IL-2Rβ alone in mouse models of resistant melanoma and pancreatic cancer [132].

### The orchestration of signaling elements within CARs

Recent data have shown that CAR signaling diverges from that of native TCR pathways and is potentially even distinct among various CAR constructs [133]. Endogenous TCRs recognize peptide–MHC complexes through a highly integrated network, and it is still unclear whether CAR engagement relies entirely on the same intracellular machinery. CARs detect antigens and initiate T-cell activation, implying that they are capable of recapitulating the three canonical types of T-cell activation: signal 1 (TCR-downstream kinase activity), signal 2 (costimulation) and signal 3 (cytokine). Above all, we separately discuss strategies to engineer these signals into the CAR architecture. Signal 1 is initiated by Lck and Src family tyrosine kinases after antigen binding; signal 2 achieves optimal activation with additional costimulatory effects to prevent anergy; and signal 3, which is typically delivered by cytokines, is considered essential for durable CAR-T-cell function [134].

To dissect how intracellular modules shape these signals, researchers recently generated a combinatorial library spanning first-, second- and third-generation CARs that incorporated various domains (CD28, 4-1BB, CD40, CTLA-4 and the IL-15Rα cytoplasmic tail) [135]. Through repeated antigen-stimulation assays, pooled functional screens and single-cell sequencing, they reported that CD28 produced strong yet transient activation, whereas 4-1BB favored an effector-memory phenotype with greater persistence. Contrary to earlier expectations, the IL-15Rα segment provided minimal additional costimulation, whereas CD40 consistently yielded the most potent and durable responses [135]. These results highlight the modularity of signaling domains; by rearranging them, investigators can further refine CAR constructs for improved therapeutic performance.

### CAR-T-cell therapy resistance

CAR-T-cell therapy dominated the cell-based immunotherapy landscape, outpacing the TCR-T, NK/NKT, and dendritic-cell (DC) approaches. By 2022, of the 1,432 active CAR-T studies, 864 were preclinical, 314 were phase I, and 243 were phase II [136]. The target choice mirrors the disease type: hematologic cancers use CD19, BCMA, and CD22, whereas solid-tumor programs focus on tumor-associated antigens such as HER2 and MSLN [136]. ClinicalTrials.gov listed nearly 1,800 cell therapy trials overall; 857 involved CAR-T cells and were directed mainly at blood cancers, whereas only approximately 43% of all cell therapy trials targeted solid tumors. Although CAR-T-cell therapy is transformative, major obstacles remain. Up to 30–70% of patients receiving CD19 CAR-T cells relapse, often after the tumor is downregulated or when CD19 is lost [137]. Toxicities, particularly cytokine-release syndrome (CRS) and immune-effector-cell–associated neurotoxicity syndrome (ICANS), further limit their use. Severe CRS (grade ≥ 3) occurs in approximately 23–46% of patients and is driven by the explosive in vivo expansion of CAR-T cells [138]. Because IL-6 blockers do not mitigate this neurotoxicity, safer CAR designs and alternative countermeasures are urgently needed. In solid tumors, a dense stroma and highly immunosuppressive microenvironment hinder CAR-T-cell trafficking and infiltration, creating an additional barrier to efficacy.

Compared with CAR-T cells, TCR-T cells exhibit greater sensitivity, allowing T cells to respond to just one or a few antigen molecules on a target cell [88]. In addition, T-cell cytotoxicity is gentler than that of CAR-T cells and triggers less cytokine release. However, tumors can evade immune surveillance by downregulating surface MHC/HLA molecules, and sourcing high-affinity TCRs remains challenging. Together, these hurdles limit the clinical efficacy of TCR-T-cell therapy and prompt the development of TCR-like synthetic receptors.

## Rising TCR-like synthetic receptors and therapy

**Fig. 3:**
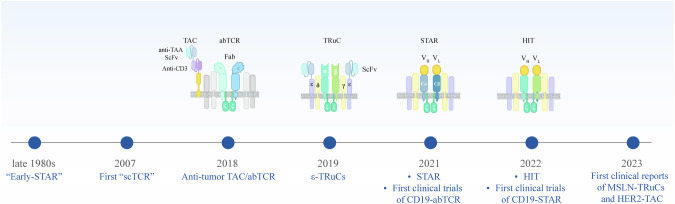
Timeline of the development of TCR-like synthetic receptors. Early designs replaced TCR Vα and Vβ with immunoglobulin variable domains but required two separate vectors, limiting expression efficiency, named “Early STAR”. The original structure of TRuC was named “scTCR”. Recently, T-cell antigen coupler (TAC), TCR fusion construct (TRuC), antibody TCR (abTCR), synthetic TCR and antigen receptor (STAR), and HLA-independent T-cell receptor (HIT) reintroduced the TCR-CD3 domain, increasing sensitivity and antitumor efficacy, particularly against solid tumors

In the late 1980s, Dr. Kuwana and Gross pioneered TCR-like constructs by swapping the TCR Vα and Vβ domains with the VH and VL antibody chains [68]. However, these early designs required separate vectors and cotransfection, which hampered transfection efficiency and unbalanced chain expression. Dr. Eshhar later introduced the single-chain CAR structure based on scFv, which quickly became the most widely researched and applied structure [5]. Recently, five separate groups reconstructed chimeric receptors, including the TAC, TruC, abTCR, STAR and HIT receptors, on the basis of the TCR-CD3 complex. In studies involving solid tumors, these synthetic receptors demonstrated superior antitumor efficacy compared with conventional CARs, revitalizing interest in more TCR-like receptor designs (Fig. 3).

### T-cell antigen coupler (TAC)

The TAC receptor comprises three domains: (1) an antigen-binding domain that recognizes the target antigen; (2) a CD3-binding domain that stably couples TAC to the endogenous TCR-CD3 complex; and (3) a CD4 coreceptor domain (including a hinge, transmembrane and intracellular domain) that helps anchor the receptor at the cell surface and recruits an additional Lck kinase [139]. Although the TAC receptor itself lacks an intrinsic signaling motif, it induces signaling by promoting TCR-CD3 polymerization. The CD4 coreceptor domain further strengthens this response by enhancing Lck recruitment. In both CD19^+^ hematopoietic and HER2^+^ solid tumor models, TAC-T cells outperform conventional CAR-T cells in antitumor efficacy, exhibit reduced cytokine release and infiltrate tumors rapidly. Currently, TAC-T cells that target CD19, HER2, and Claudin18.2 have been developed, and TAC-T cells that target HER2 (ERBB2) have progressed to phase I/II clinical trials [140, 141].

### TCR fusion constructs (TRuC)

TruC fuses a scFv directly to one of the five TCR-CD3 subunits (TCRα, TCRβ, CD3γ, CD3δ, or CD3ε), reprogramming an intact TCR complex to recognize tumor antigens [142]. When the scFv binds to its target antigen, it triggers TCR-CD3 polymerization and initiates downstream T-cell signaling. Researchers have avoided linking the scFv to CD3ζ because elongating its short extracellular domain can disrupt TCR assembly [143]. Attaching the scFv to TCRα or TCRβ could lead to mispairing with endogenous TCRβ or TCRα, potentially reducing surface display. In contrast, the fusion of scFv to CD3ε (ε-TruC) achieves robust expression and outperforms conventional CAR-T cells against lymphoma, multiple myeloma and solid tumors [142, 144, 145]. This enhanced efficacy may result from TRuC activating the full TCR signaling program, including the phosphorylation of CD3ε and CD3ζ, which is not observed in CAR-T cells [142, 146]. Consequently, ε-TRuC T cells display reduced cytokine release and exhaustion, more efficient tumor infiltration, and improved persistence in solid tumor models. Researchers have further optimized ε-TRuCs by adding CD28 or 4-1BB costimulatory domains, coexpressing IL-7 and incorporating regulatory fusion proteins such as PD1-CD28 [147–149]. These modifications could promote the function of the original ε-TRuC T cells to different degrees. Other modifications include substituting the Vα or Vβ of the TCR with the scFv (TCAR1) and fusing the scFv to the constant region of the TCRγ or TCRδ chain (γ-TCRγδ or δ-TCRγδ) [150, 151]. In one study, introducing CD19 ε-TRuC into γδ T cells expanded by zoledronate or concanavalin A improved tumor cell recognition, cytotoxicity and cytokine production [152]. Moreover, a bispecific TRuC that targets BCMA and CD2 subunit 1 (CS1) for treating relapsed/refractory multiple myeloma, which is also engineered to secrete IL-7 and CCL21, demonstrated enhanced antitumor potency and persistence [153]. TCR^2^ therapeutics reported clinical data for Gavo-cel, a mesothelin (MSLN)-targeted TRuC T-cell therapy for advanced solid tumors. Approximately 93% of treated patients experienced tumor shrinkage, with an objective response rate (ORR) of 22%. Among these heavily pretreated patients, 70% survived at least six months, and 31% reached one-year survival [145].

### Antibody TCRs (AbTCRs)

The AbTCR fuses the variable heavy (VH) and variable light (VL) regions of an antibody into the constant regions derived from the γδTCR [154]. The VH and VL moieties recognize antigens, whereas the Cγ and Cδ subunits couple with CD3 subunits, enabling intracellular signaling. Because Cγ/Cδ does not interact with the α/β TCR chains, abTCR prevents the mispairing of other chimeric receptors and maintains stable surface expression. Like TAC and TRuC, CD19 abTCR-T cells exhibit less exhaustion, a stronger memory phenotype, lower cytokine release, and improved antitumor efficacy in preclinical models. With this approach, researchers replaced the abTCR extracellular region with a TCR-mimic antibody that recognizes intracellular antigens presented by the MHC and adds a second chimeric receptor to bind tumor cell membrane proteins and provide costimulation [155]. In hepatocellular carcinoma, the abTCR recognizes alpha-fetoprotein presented by HLA-A*02, while a second chimeric receptor, consisting of a GPC3-specific scFv fused to CD28, delivers additional stimulation [156]. Similarly, an ESK2 TCR-mimic antibody specific for Wilms tumor protein (WT1) presented by HLA-A*02 was coupled with a CD33-specific scFv (linked to CD28), demonstrating promising therapeutic effects against AML in vitro and in animal models [155]. In a first-in-human trial (NCT04014894), eight patients with relapsed or refractory diffuse large B-cell lymphoma (R/R DLBCL) received CD19-abTCR T cells. Seven patients achieved clinical responses, and six patients achieved complete response (CR), with only one patient experiencing ICANS of Grade 3 [157, 158].

### Synthetic TCR and antigen receptor (STAR)

More recently, our group developed STAR constructs by fusing VH and VL separately to murine-mutant TCRα and TCRβ constant regions, thus avoiding mispairing with endogenous human TCR chains [159]. Unlike abTCR, which uses γδ TCR chains, and TAC or TruC, which employs linear scFvs, the STAR structure most closely resembles a native TCR in both size and construct but does not require CD8 or CD4 coreceptors because it recognizes antigens in an MHC-independent manner [160]. Compared with conventional CAR-T cells, STAR-T cells exhibit lower spontaneous activation, reduced exhaustion and faster tumor infiltration in multiple preclinical models [159]. STAR-T cells also demonstrate greater sensitivity toward tumor cells with lower antigen abundance and express fewer immunosuppressive molecules, resulting in enhanced antitumor efficacy in vivo [159]. For further development, STAR receptors were designed with two different scFvs to achieve dual targeting and incorporate costimulatory [161], cytokine receptor or fusion proteins [159, 162].

In clinical settings, single-target CD19/STAR-T (NCT03953599) [163] and dual-target CD19/CD20 STAR-T (NCT04260945) have shown promising efficacy and safety in investigator-initiated trials (IITs), both of which received IND approvals, initially confirming the efficacy and safety of STAR therapy. LILRB4-STAR T cells (NCT05548088) were tested in patients with relapsed/refractory AML, yielding a 50% overall response rate (3 out of 6), with two responders successfully undergoing subsequent allo-HSCT without minimal residual disease (MRD). Additionally, a trial evaluating CD19 STAR T cells in patients with recurrent/refractory autoimmune diseases (NCT06379646) suggested potential utility beyond cancer immunotherapy.

### HLA-independent T-cell receptors (HITs)

In 2022, Dr. Sadelain’s group fused the variable region of the light and heavy chains of an antibody directly to the constant region of human TCRα and TCRβ, creating HIT-T cells [164]. To prevent mispairing with endogenous TCRαβ chains, the constructs were inserted into the *TRAC* locus via gene editing. Like STAR T cells, HIT T cells display greater antigen sensitivity than conventional CAR-T cells do [159, 164]. Even without additional costimulatory domains, HITs exhibit antitumor efficacy comparable to that of CD28ζ CAR-T cells. When coexpressing CD80 and 4-1BBL, HIT-T cells show greater proliferation and survival than CD28ζ CAR-T cells [164, 165]. Like TRuCs and STAR, HIT-T cells exhibit decreased exhaustion, enhanced persistence and better tumor infiltration into solid tumors than CAR-T cells [142, 159, 164]. However, because the *TRBC* locus was not edited, endogenous TCRβ could pair with the synthetic TCRα chain, resulting in reduced surface expression and limiting its efficacy. However, HIT-T cells might be particularly promising against solid tumors or tumor cells with lower antigen abundance, which often evade conventional CAR-T-cell therapies.

## Comparison of CAR and TCR-like synthetic receptors

### Structure and signaling

TCR-like synthetic receptors, including STAR, HIT, TRuC, abTCR and TAC, depend on the endogenous TCR-CD3 complex to transmit activation signals. In contrast, conventional CARs self-oligomerize on the cell surface and include a CD3ζ domain for signaling. Because a single TCR-CD3 complex provides up to 10 ITAMs, versus only 3 ITAMs in a typical CAR, TCR-like receptors can potentially activate T cells with greater strength and sensitivity, especially under conditions of low antigen density. Research has shown that minimal ITAM phosphorylation (approximately 2 to 4) triggers cytokine secretion, whereas full phosphorylation of 10 ITAMs is necessary for robust T-cell proliferation [77]. Stronger activation signals broaden each T-cell’s functional response and activate more cells overall, leading to a more effective immune response [166]. Unlike classical TCRs, however, constructs such as STAR or HIT do not require CD8 or CD4 coreceptors, as they recognize antigens in an MHC-independent pattern [160, 164]. As reported, TCR-like synthetic receptors often form synapses resembling those of native TCRs, with F-actin enrichment and the accumulation of LAMP-1 lysosomes [164]. CAR molecules, however, generate more disorganized synapses that rapidly dissociate [167]. Furthermore, owing to their unique single-chained structure, CARs can spontaneously cluster in the absence of antigens (tonic signaling), accelerating T-cell differentiation and exhaustion while impairing antitumor effects [88, 168, 169]. Recently, Dr. Haopeng Wang revealed that positively charged patches (PCPs) in the CAR antigen-binding domain can lead to CAR clustering and tonic signaling, introducing the AI-driven tool “CAR-Toner” to predict and mitigate tonic signaling in CAR-T cells [170, 171]. TCR-like receptors do not exhibit tonic signaling, which might explain their reduced exhaustion and more naïve cell phenotype [142, 159, 164].

Second-generation CARs incorporate costimulatory domains (e.g., CD28 or 4-1BB) to enhance effector function and promote T-cell persistence via the noncanonical NF-κB pathway [133, 172]. In contrast, original TCR-like receptors induce TCR signaling (signal 1) but lack costimulatory signaling (signal 2). Despite this, STAR and HIT-T cells exhibit comparable effector functions as second-generation CAR-T cells in several tumor models [159, 164]. Additional modifications in TCR-like receptors, such as the fusion of costimulatory, cytokine or fusion proteins, further increase T-cell function to different degrees [149, 162]. Although both TCR-like receptors and CARs rely on immunoglobulin-based domains for antigen binding, differences in the extracellular regions also shape T-cell activation. The native TCR-pMHC intercellular space is approximately 14 nm long, which is optimal for the formation of immunological synapses. In contrast, the hinge length in CARs can vary widely, affecting synapse organization and T-cell function [173–176].

### Effector function

T lymphocytes exert killing functions in several different ways. First, the effector synapse formed between cytotoxic T lymphocytes and target cells serves not only as a signaling amplifier but also as a channel that bridges the two cell types and transmits cytolytic vesicles containing perforin and granzyme [177]. Perforin polymerizes to form a tubular structure inserted into the cell membrane, where granzymes enter the target cell and initiate serine protease activity, leading to the cleavage of proteins such as caspases and gasdermin, thus activating cell death pathways such as apoptosis or pyroptosis [178]. Second, T cells induce target cell apoptosis via the Fas‒FasL axis: soluble FasL from T cells can induce the trimerization of Fas molecules on target cells [179]. Trimerized Fas activates caspase-8 through the adapter protein FADD, and caspase-8 further cleaves and activates caspase-3 to initiate apoptosis [180]. Moreover, cytokines secreted by T cells enhance tumor cell sensitivity to death-inducing factors. High concentrations of TNF-α are able to induce the apoptosis and necrosis of tumor cells by binding to TNFR1/2 on the tumor cell surface, whereas IFN-γ and TNF-α can inhibit the expression of Bcl-XL in tumor cells and thus increase the susceptibility of tumor cells to TRAIL-mediated apoptosis [181]. Moreover, in the tumor microenvironment, IFN-γ can help control tumors by activating other immune cells, including promoting the differentiation of macrophages into the M1 subtype [182].

TCR-like receptors (STAR, HIT, TRuC, AbTCR, and TAC) and CARs all show considerable killing ability toward cells expressing cognate targets. However, TCR-based constructs exhibit 10- to 100-fold greater sensitivity despite lower surface expression. This heightened sensitivity is especially evident against target cells with low antigen density, where STAR/HIT-T cells typically outperform CAR-T cells utilizing the same recognition scFv [164]. This discrepancy may arise from distinct immunological synapse architectures. In addition to cytotoxicity, cytokine production also dictates the effector function of T cells. In vitro, STAR-T cells produce more TNFα, IFNγ and IL-2 than CAR-T cells [159]. However, in vivo, TCR-like receptor (STAR, TRuC, TAC and AbTCR) T cells often release fewer cytokines/GMCSF than CAR-T cells [139, 142, 154, 159]. Persistence, which is the ability to maintain function under chronic antigen exposure, is another crucial aspect of T cells. As discussed previously, CAR-T cells can undergo “tonic signaling” even without antigen stimulation, leading to premature T-cell exhaustion; in contrast, TCR-like receptors remain in a quiescent state until antigen engagement, retaining greater effector potential [88]. After being exposed to antigens, T cells engineered with TCR-like receptors showed a significantly stronger killing ability after days of repeated antigen stimulation, which demonstrated that these constructs entitle T cells with greater persistence than the CAR construct. Moreover, TRuC, STAR and HIT-T cells also persist longer and infiltrate into solid tumors better than CAR-T cells [159, 164, 165]. The in vivo results are an integrated outcome of these characteristics. Overall, by leveraging native TCR signaling and a more balanced activation profile, TCR-like receptors can offer advantages over conventional CAR constructs.

### Potential limitations

Although TCR-like receptors offer clear advantages, they also inherit some constraints from the native TCR complex. Antigen-binding triggers rapid internalization of the TCR—an essential “brake” that prevents overstimulation but simultaneously weakens signaling in engineered T cells [183]. STAR and HIT receptors, for example, assemble with CD3 subunits in endosomes before being displayed on the membrane. Mutations that slow or reduce TCR–CD3 internalization may therefore increase their functional persistence. CAR molecules, which are independent of CD3 subunit assembly, may share different internalization mechanisms. Upon binding to tumor antigens, CARs are rapidly ubiquitinated, triggering their internalization and lysosomal degradation. Replacing all lysine residues in the cytoplasmic domain (CAR^KR^) blocks this ubiquitination, promotes recycling to the cell surface, and enhances antitumor activity [93]. Because TCR-like receptors pair with endogenous TCR subunits, they are displayed on the cell surface at lower densities than most CARs are, yielding weaker signaling and minimal tonic signaling. Carefully tuned tonic signaling enhances T-cell fitness, suggesting that similar adjustments could benefit TCR-like platforms. In support of this idea, researchers recently engineered T cells that coexpress a native TCR and a CAR [184]. Strong TCR–antigen interactions amplified CAR signaling, whereas weak interactions attenuated it, producing cells with superior antitumor potency and reduced off-target toxicity. Further structural modifications are necessary to balance tonic and ligand-induced activation of both TCR-like receptors and next-generation CARs.

## Perspective

### Cellular modification: gene editing

Despite the above improvements in the use of synthetic receptors to enhance T-cell function, the limitations of CAR-T-cell therapy are multifactorial and cannot be solved by a single approach. With the development of clustered regularly interspaced short palindromic repeats (CRISPR) and CRISPR-associated protein 9 (Cas9), addressing limitations in T-cell function via gene editing has become easy. The CRISPR/Cas system was first identified as an adaptive immune mechanism in prokaryotes that provides defense against viruses and plasmids [185, 186]. The designed guide RNA (gRNA) could direct the Cas9 DNA endonuclease enzyme to a specific location in the DNA sequence. Once there, Cas9 creates a double-strand break in the DNA, allowing for the insertion, deletion, or modification of genetic material. These approac.hes have been applied for immunotherapy, including the direct delivery of therapeutic vectors to specific gene loci, the knockout of negative regulators of T-cell function, or the generation of safe and potent allogenic universal CAR-T-cell products [187–189].

Currently, engineered T cells, which can randomly integrate into the host genome, are typically generated via lentivirus/retrovirus delivery. This random integration raises concerns about the potential for uncontrolled cell growth, and risk tends to become a tumor. Therefore, one application of gene editing is to target integration into specific genome loci, which can ensure safe and more consistent expression, such as when the CD19 CAR is integrated into the *TRAC* locus [190, 191]. Directly integrating CARs into the native genetic locus of the TCR complex may enhance the efficacy of CAR-T cells, likely due to the more advantageous expression dynamics provided by the TCR promoter.

The use of CRISPR-Cas9 to target inhibitory receptors such as PD-1 to eliminate negative regulators of T-cell effector function is another tractable point. Recently, Dr. He Huang’s group generated nonviral, *PD1*-integrated CD19 CAR-T cells via CRISPR-Cas9. A preclinical study revealed that these engineered CAR-T cells demonstrated high efficacy in eradicating tumor cells and achieved an 87.5% complete remission rate in eight patients with relapsed/refractory aggressive B-cell non-Hodgkin lymphoma without serious adverse events (NCT04213469) [192]. In addition to the use of inhibitory receptors, removing genes encoding cytokines such as GM-CSF, which has the potential to drive clinical CRS or neurotoxicity with CRISPR-Cas9, results in safe, potent and durable cell products [193]. Compared with conventional CD19 CAR-T cells, CD19 CAR-T cells with GM-CSF knockout maintained normal functions, had enhanced antitumor activity and improved overall survival [193]. In clinical trials, especially for some newborn or elderly patients, it is difficult to obtain enough high-quality T cells to generate patient-specific engineered T cells, and CRISPR-Cas9-mediated multiplex gene editing to develop “universal” CAR-T cells from healthy donors may be used to treat different patients [194]. Ideally, universal T-cell products need to be resistant to natural killer (NK) cells and T cells from the host and overcome the induction of graft-versus-host disease (GvHD), as endogenous donor T cells recognize “nonself” surface human leukocyte antigen (HLA) on patients, eliciting immune attack. A previous study illustrated a strategy involving the use of CRISPR-Cas9 to create universal CAR-T cells [194]. *TRAC*, *B2M*, and *PD-1* genes were simultaneously knocked out to eliminate T-cell receptor expression and reduce immunogenicity, thereby preventing GvHD. The engineered CAR-T cells demonstrated effective antitumor activity in vitro and in vivo, maintaining their potency against CD19-expressing tumors [194]. The safety and feasibility of this approach have been proven by the first-in-human trial of multiple gene editing approaches (NCT03399448) [195]. In this study, researchers disrupted the *TRAC*, *TRBC*, and *PD1* genes to enhance antitumor immunity and introduced a cancer-targeting transgene, NY-ESO-1. The engineered T cells were well tolerated, showed durable engraftment for at least nine months, and demonstrated specific antitumor activity, with reduced target antigens in patients with myeloma [195]. Recently, Dr. Huji Xu reported a study in which CRISPR-engineered allogeneic CAR-T cells targeting CD19 were used to treat patients with refractory autoimmune diseases (NCT05859997). The genetically modified T cells were infused into the patients, which persisted for more than three months and achieved complete B-cell depletion within two weeks. During a six-month follow-up, all patients experienced significant clinical improvements without serious adverse events, indicating the safety and potential efficacy of off-the-shelf CAR-T cells in managing severe autoimmune conditions in addition to tumors [196]. In conclusion, CRISPR/Cas9 gene editing technology provides a powerful tool to address some of these limitations by allowing for targeted modifications in T cells. In addition, it enables precise editing of genetic material, facilitating the creation of more effective and safer engineered T-cell products. Ongoing research efforts are attempting to address the complexities and variabilities associated with this technology to ensure comprehensive, effective, and safe applications.

### Potential applications beyond tumors

**Fig. 4:**
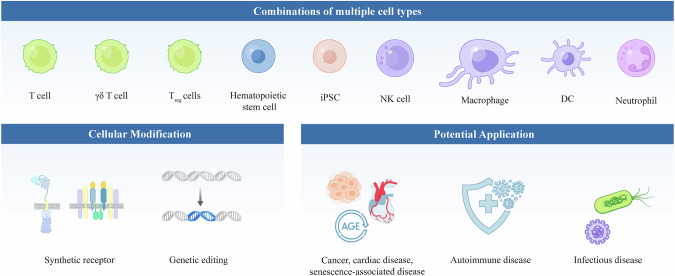
The future of engineered immune cell therapy. Overview of next-generation immunotherapy, which involves diverse engineered immune cells—T cells, NK cells, macrophages, dendritic cells and neutrophils from patient or iPSC sources—combined with CRISPR/Cas9 gene editing to address multifactorial CAR-based therapy challenges and expand treatment options beyond cancer to include autoimmune, cardiac, senescence-associated, and infectious diseases

As discussed above, engineered T cells have revolutionized the field of immunotherapy for other diseases, such as autoimmune diseases, cardiac diseases, senescence-associated diseases and infectious diseases [197–199] (Fig. 4). Inspired by CAR-T-cell therapy to treat cancers, chimeric autoantibody receptor (CAAR) has been developed to mitigate pathogenic antibodies in systemic lupus erythematosus (SLE) and pemphigus vulgaris (PV) [200–202]. CAAR-T cells for treating PV, which consists of the PV autoantigen desmoglein (Dsg)3, can specifically eliminate autoreactive B cells expressing anti-Dsg3 B-cell receptors, resulting in potent cytotoxicity in vitro and in vivo without off-target effects. CAAR-T cells persist and effectively reduce serum autoantibody levels and disease symptoms; currently, a clinical trial is ongoing (NCT04422912) [200]. For SLE, CD19 CAR-T cells are utilized to deplete circulating B cells completely, leading to a decrease in anti-dsDNA antibodies and proteinuria, which significantly improve in different patients [201, 202]. In addition, short-term follow-up indicates that although naïve B cells reappear a few months after CAR-T-cell infusion, there are no apparent disease symptoms [201, 202]. Recently, researchers evaluated the safety and efficacy of CD19 CAR-T-cell therapy in 15 patients with severe autoimmune diseases, including SLE, idiopathic inflammatory myositis, and systemic sclerosis (MS). Following a single infusion of CAR-T cells after lymphodepletion, all patients achieved remission, with significant improvements in disease activity scores. The treatment was well tolerated, with mild CRS observed. Notably, patients are able to discontinue immunosuppressive therapy without relapse [203]. BCMA-CAR-T cells for treating neurological autoimmune diseases are promising. Recently, researchers explored the effects of BCMA-CAR-T-cell therapy in patients with neuromyelitis optica spectrum disorder (NMOSD) through single-cell multiomics analysis of blood and cerebrospinal fluid samples. Proliferating CD8^+^ CAR-T-cell clones are key players in autoimmunity, demonstrating their enhanced chemotactic abilities and reduced cytotoxicity [204].

In addition, engineered T cells have been used to treat cardiac disease. Owing to the identification of fibroblast activation protein (FAP) as a promising target for the treatment of solid tumors [205], engineered CAR-T cells that recognize FAP also significantly reduce cardiac fibrosis and improve heart function in the treatment of myocardial diseases [206]. Similarly, a novel approach to generate transient FAP CAR-T cells in vivo involves the use of CD5-targeted lipid nanoparticles to deliver modified mRNA, enabling the production of CAR-T cells directly within the body to treat cardiac fibrosis [207]. Senescence-associated diseases are characterized by the accumulation of senescent cells, which are damaged or dysfunctional cells that have lost the ability to divide but remain metabolically active [208]. These cells can contribute to chronic inflammation and tissue dysfunction, leading to various age-related diseases, including cardiovascular diseases, neurodegenerative disorders, and autoimmune conditions [209]. Evidence has shown that targeting senescent cells, such as senolytics or immunotherapies such as CAR-T cells, aims to mitigate their detrimental effects and improve health outcomes in aging populations [210, 211]. Recently, researchers developed urokinase-type plasminogen activator receptor (uPAR) CAR-T cells that effectively eliminate senescent cells in fibrotic tissues in a mouse model, leading to improved liver function and reduced fibrosis, suggesting a novel approach to enhance tissue regeneration and combat age-related diseases through engineered T cells. The therapeutic potential of these CAR-T cells is to treat senescence-associated diseases, including liver fibrosis, and potentially other conditions, such as atherosclerosis and diabetes [212]. In addition, CAR-T cells targeting uPAR^+^ senescent cells can alleviate metabolic dysfunction [213]. In mouse models, researchers have reported that these CAR-T cells effectively reduce the burden of senescent cells in various tissues, improving glucose homeostasis and metabolic fitness without causing toxicity [213]. Strategies for treating infectious diseases, such as HIV/AIDS, are also being explored. Several clinical trials have investigated the safety and efficacy of using CD4ζCAR-T cells in HIV-infected individuals between 1995 and 2005 [214, 215] Along with the development of first- to second-generation CARs, optimized CD4-based CAR-T cells containing the 4-1BB–CD3ζ signaling domain were at least 50-fold more potent at suppressing HIV replication than were T cells expressing the original CD4ζCAR [216]. In addition, researchers have explored targeting HIV-infected cells with alternative antigen-binding moieties, such as scFvs derived from broadly neutralizing antibodies (bNAbs), which target conserved sites within the Env protein [217]. CAR-T cells have also been developed for fungal infections. *Aspergillus fumigatus* is a fungus responsible for invasive pulmonary aspergillosis (IPA), particularly in immunocompromised patients. Researchers have engineered Af-CAR-T cells that specifically recognize *A. fumigatus hyphae*, demonstrating their ability to exert direct antifungal effects and activate macrophages to enhance the immune response [218] In summary, engineered T cells have substantially transformed the landscape of immunotherapy, extending their therapeutic applications beyond cancer to include various diseases, such as autoimmune disorders, cardiac conditions, senescence-associated diseases, and infectious diseases. Despite these advancements, several limitations remain. The long-term efficacy and safety of engineered T-cell therapies necessitate careful evaluation across diverse patient populations. Future research should focus on refining engineering processes, exploring novel targets, and enhancing the safety profiles of engineered T cells to ensure their effectiveness in clinical settings.

### Combinations of multiple cell types

Currently, engineered cell therapy involves multiple immune cell types, including T cells, natural killer (NK) cells, macrophages, and dendritic cells derived from autologous patient-derived or induced pluripotent stem cells (iPSCs) (Fig. 4). In 2022, 2756 active cell therapies, including CAR/TCR-T cells, NK/NKT cells, dendritic cells, myeloid cells and stem cells, were recorded. Among the various categories, CAR-T-cell therapy and NK-cell therapy increased by 24% and 55%, respectively, which are smaller increases than those reported in 2021 [219]. Other cell therapies, such as the use of dendritic cells, stem cells or myeloid cells, among others, grew by 129% this year, a rapid increase compared with the 37% growth rate between 2020 and 2021 [219]. Promising studies on conventional T cells have raised the prospect of developing engineered cell therapies involving other immune cell types to combat cancer, autoimmune disorders and more.

γδ T cells, which express a heterodimeric TCR composed of γ and δ chains, represent a unique subset of T lymphocytes. Unlike αβT cells, γδ T cells are not constrained by classical HLA–peptide complexes; instead, they recognize a wide range of stress signals and metabolic changes induced by infection or malignancy [220, 221]. This makes γδ T cells inherently allogeneic-friendly, as they can be transferred between individuals with minimal risk of graft-versus-host disease. Circulating γδ T cells are dominated by the Vγ9/Vδ2 subset, which exerts potent antitumor functions by secreting inflammatory cytokines such as IFN-γ, TNF-α, and IL-17. Notably, IL-17-producing γδ T cells (Vδ1) can synergize with immunogenic cell death-inducing chemotherapeutic drugs, fueling robust antitumour immunity [222]. Over the past decade, strategies to expand and engineer γδ T cells have matured significantly. To expand multiple γδ T-cell subsets and engineer them to express a CD19 CAR, researchers have used the sleeping beauty transposon system and a K562-based artificial antigen-presenting cell to stably introduce and drive the proliferation of polyclonal γδT cells, preserving diverse Vγ and Vδ subsets [223]. These genetically modified cells demonstrated enhanced cytotoxicity against CD19^+^ tumor targets and significantly reduced leukemia burden in vivo. γδ T cells can also cross-present tumor antigens to αβ T cells, functioning as antigen-presenting cells while retaining intrinsic tumor reactivity [224]. Recent efforts have further refined γδ T-cell engineering to mitigate on-target/off-tumor toxicity. For example, by fusing an anti-GD2 ectodomain to a DAP10 costimulatory domain, CAR activation becomes conditional on simultaneous γδTCR signaling, ensuring aggressive tumor killing without damaging healthy tissue [225]. These engineered γδT cells can be expanded from peripheral blood and show strong antitumor activity, moderate IFN-γ production, and potential for improved safety [225]. Compared with conventional T-cell-engineered CARs, the advantages of generating CD19-CAR γδ T cells include both CAR dependence toward CD19^+^ cells and natural γδT-cell cytotoxicity against CD19^-^ leukemia cells [226]. These advances highlight the versatility of γδ T cells, positioning them as promising platforms for allogeneic cell therapies that combine robust antitumor functions with minimal safety concerns.

Engineered regulatory T cells (Tregs) represent a promising alternative for treating autoimmune disease, transplant rejection and graft-versus-host disease [227, 228]. Current engineering strategies aim to increase Treg functionality, stability, trafficking, and persistence in vivo. Early investigations into CAR-Tregs highlighted their potential in transplantation scenarios. For example, researchers developed Tregs that express a humanized HLA-A2-specific CAR (A2-CAR), assessing their ability to traffic to target tissues, suppress immune responses, and promote allograft tolerance [229, 230]. In xenogeneic GvHD and transplant models involving islet and human skin, these CAR Tregs have been shown to effectively mitigate immune-mediated damage, setting the stage for clinical evaluation [229, 230]. Two clinical trials (NCT04817774 and NCT05234190) are currently assessing the safety and efficacy of A2-CAR-T cells in kidney and liver transplant recipients. Additionally, researchers are refining CAR design to optimize Treg performance through various intracellular signaling enhancements. Comparative studies have evaluated A2-CAR-T-cell constructs featuring different costimulatory domains, such as CD28, 4-1BB, ICOS, and OX40, to determine the most effective configurations for immunosuppressive therapy [231]. Notably, in a xenogeneic GVHD disease model, Tregs with a wild-type CD28 CAR demonstrated superior survival, proliferation, and suppressive capabilities, emphasizing that the optimal signaling requirements for Tregs differ substantially from those of conventional T cells. Further studies have indicated that CAR constructs incorporating CD28 costimulatory domains yield better results than those containing 4-1BB domains in enhancing Treg functionality [232, 233]. Recently, researchers have explored the overexpression of Foxp3 in CD19 CAR-T cells, which specifically target B cells that contribute to lupus pathology. This modification resulted in enhanced Treg function, phenotype stability, and immunosuppressive capacity both in vitro and in a humanized mouse model of systemic lupus erythematosus (SLE) [233]. Importantly, these CAR-Tregs do not eliminate B cells; rather, they selectively moderate pathogenic B-cell clones while preserving nontargeted B-cell populations. This dual mechanism facilitates broader immunomodulatory effects, allowing CAR-Tregs to influence T-cell populations within affected tissues and promoting a more balanced immune response during disease conditions [233]. Overall, the engineering of Tregs, particularly through CAR technology, opens a new frontier in therapeutic interventions aimed at regulating the immune response and enhancing transplant acceptance, ultimately paving the way for improved management of autoimmune disorders, transplant outcomes, and GvHD.

In addition to T cells, engineered cell therapies based on other immune cell types, particularly natural killer (NK) cells and macrophages, have shown significant clinical potential. As part of the innate immune system, NK cells utilize a combination of activating and inhibitory receptors to identify and target tumor cells effectively [234–236]. In a promising phase 1/2 trial (NCT03056339), researchers developed allogeneic CD19 CAR-NK cells derived from cord blood to treat patients with relapsed or refractory non-Hodgkin’s lymphoma or chronic lymphocytic leukemia (CLL). Among the 11 heavily pretreated participants, eight demonstrated a positive response, seven of whom achieved complete remission. Notably, this therapy exhibited minimal toxicity, with no observed cases of cytokine release syndrome, neurotoxicity, or graft-versus-host disease. The engineered NK cells expressing IL-15 and an inducible caspase 9 safety switch demonstrated in vivo expansion and persistence for up to 12 months [237]. While the therapy was generally well tolerated, the long-term durability of responders remains uncertain, as some patients received subsequent treatments. In another phase I trial for metastatic colorectal cancer (NCT03415100), locally administered NKG2D CAR-NK cell therapy resulted in a reduction in malignant ascites in two patients and a complete metabolic response in a liver lesion in a third patient, all without major adverse events [238]. Recent advances have focused on enhancing NK cell function through genetic modifications. For example, researchers combined the genetic removal of cytokine-inducible Src homology 2-containing (CIS), a negative regulator of IL-15, with “armored” CAR engineering in cord blood-derived NK cells. By deleting the CISH gene, IL-15 signals intensify, enhancing NK cell function via the Akt/mTORC1 pathway and c-MYC, thus increasing aerobic glycolysis and cytotoxicity. Combining IL-15 secretion and CIS blockade has been shown to be more effective than either modification alone [239]. These findings underscore the potential of transient CAR expression on NK cells for creating safe and potent cancer immunotherapies.

As professional phagocytes and integral components of the innate immune system, macrophages also present unique engineering opportunities. Immunosuppressive tumor-associated macrophages (TAMs), often referred to as M2 macrophages, can suppress T-cell responses and promote tumor progression. Conversely, M1 polarization is characterized by a proinflammatory phenotype that exhibits antitumor activity, generating significant interest in engineering macrophages for cancer therapy to enhance immune surveillance. Previous studies reported that CARs for phagocytosis (CAR-Ps) are engineered to direct macrophages to engulf cancer cells. CAR-Ps consist of an antibody fragment that targets specific antigens, enhancing macrophage phagocytosis. These findings demonstrate that CAR-P-expressing macrophages can effectively engulf antigen-coated particles and reduce cancer cell numbers by more than 40% in coculture [240]. Recently, in another study, CAR-Ms were used to target cancer cells, specifically in a lung metastasis model in which SKOV3 cells were used [241]. The results showed that CAR-M-cell therapy significantly reduced the tumor burden and prolonged overall survival [241]. In addition, immunohistochemical analysis confirmed a substantial decrease in metastatic tumor nests in the lungs of CAR-M-treated mice. This study also included RNA sequencing of human macrophages to identify differentially expressed genes related to macrophage function. Additionally, T-cell stimulation assays demonstrated the potential of CAR-Ms to enhance antitumor efficacy [241]. Researchers have also engineered anti-HER2 CAR macrophages (CAR-Ms) designed to target and eliminate tumor cells while actively reprogramming the tumor microenvironment [241]. These CAR-Ms promote proinflammatory phenotypes and sustain an M1-like state within the tumor microenvironment, facilitating the cross-presentation of tumor antigens to T cells and enhancing overall antitumor activity. An alternative approach is to engineer CAR-macrophages (CAR-147) to target the extracellular matrix (ECM) in solid tumors, specifically in HER2-positive breast cancer [242]. Although CAR-147 macrophages did not affect tumor cell growth in vitro, their infusion significantly inhibited tumor growth in BALB/c mice. This effect was associated with reduced collagen deposition in tumors and increased T-cell infiltration. In this construct, CAR-147 macrophages were designed to recognize the HER2 antigen, activating CD147 signaling to increase matrix metalloproteinase (MMP) expression, which aids in ECM degradation. Notably, the levels of inflammatory cytokines such as TNF-α and IL-6 are significantly lower in the blood of treated mice, suggesting a reduced risk of CRS [242]. These findings indicate that targeting the ECM with engineered macrophages could be an effective strategy for treating solid tumors, promoting T-cell activity while minimizing side effects. These findings suggest that CAR-Ms represent a promising therapeutic strategy for cancer treatment, particularly in overcoming challenges associated with solid tumors.

Dendritic cells, on the other hand, play crucial roles in priming naïve T cells and eliciting tumor-specific responses [243]. Recently, via single-cell RNA sequencing, researchers identified distinct DC subsets in naïve and tumor-bearing lungs, revealing a regulatory program that impairs DC1 functionality [243]. This program is associated with the uptake of apoptotic cell antigens and is enriched in antigen-charged DCs that migrate to draining lymph nodes (DLNs). Importantly, the absence of CD8α^+^ DCs results in reduced T-cell priming and effector function [244]. This finding highlights the importance of CD8α^+^ DCs in presenting antigens and activating cytotoxic T cells, which are vital for effective immune responses against tumors. Overall, these studies underscore the potential of DCs to enhance immunotherapy strategies for cancer treatment, which has become an emerging strategy for engineering chimeric receptors. For example, using a 4-1BB-based CAR to engineer dendritic cells, researchers have shown that these CAR-DCs can produce IL-12, enhancing the effectiveness of anti-CD33 CAR-T-cell therapy for acute myeloid leukemia (AML). The engineered CAR-DCs presented increased intratumoral dendritic cell subsets and a proinflammatory phenotype that significantly bolstered CAR-T-cell activation, resulting in increased production of IFN-γ and TNF-α. The combination of CAR-DCs with CAR-T cells not only showed superior cytotoxicity against AML cells but also prolonged survival and reduced disease burden in an NSG mouse model [245]. These findings suggest that the interaction between CAR-DCs and CAR-T cells enhances antitumor immunity, suggesting a novel strategy to improve CAR-T-cell therapy efficacy in cancer treatment.

Neutrophils are the most prevalent type of white blood cell in the bloodstream and serve as the body’s first line of defense against infections [246]. Traditionally viewed as simple phagocytes, neutrophils are now recognized for their complex functions in immune regulation and tissue repair. Neutrophils can act as double-edged swords, where their potent antimicrobial mechanisms can inadvertently cause collateral damage to host tissues, particularly in the tumor microenvironment [246]. Various phenotypes of neutrophils and their plasticity in response to different tissue cues complicate their role in cancer. It also addresses the challenges of targeting neutrophils therapeutically, as their robust and adaptable nature makes them difficult to manipulate without risking adverse effects. Recently, to treat glioblastoma (GBM), researchers engineered CAR neutrophils combined with nanodrugs. Despite the challenges of delivering therapies across the blood‒brain barrier and the short lifespan of neutrophils during preparation and administration, optimizing the preparation time and increasing the dosage of CAR-neutrophils and nanodrugs significantly improved survival rates in tumor-bearing mice [247]. R-SiO2–TPZ-loaded CAR-treated neutrophils effectively maintained their antitumor N1 phenotype and could target GBM cells in various tumor microenvironments. Combining CAR-neutrophil therapy with more effective chemotherapy agents or radiosensitizers could enhance antitumor efficacy. Additionally, extending the shelf-life of neutrophils and employing controlled drug release systems may further improve therapeutic outcomes [247]. In addition, engineered human pluripotent stem cells (hPSCs) with synthetic CARs can be differentiated into functional neutrophils that exhibit potent cytotoxicity against tumor cells both in vitro and in vivo [248]. Similarly, these CAR-derived neutrophils maintain an antitumor N1 phenotype, demonstrating superior tumor infiltration and killing capabilities compared with those of wild-type neutrophils. The scalability of this CAR-neutrophil production platform could lead to standardized cellular products for clinical applications in other cancer treatments. However, the long-term efficacy and safety of engineered CAR-neutrophil still need to be investigated in clinical settings. The use of neutrophils also includes their derivatives, such as membranes and extracellular vesicles, as advanced drug delivery systems. These cellular carriers can enhance tumor targeting and therapeutic efficacy by overcoming barriers to drug delivery. Various strategies for engineering neutrophils, such as improving drug delivery and remodeling the TME, including the use of nanomaterials and hybrid cellular membranes, need to be explored. Further research is needed to understand their complex roles and optimize therapeutic strategies. This includes profiling neutrophil subpopulations and their functions in different tumor contexts to develop effective and personalized cancer treatments. Overall, neutrophils represent a new frontier in cancer therapy, combining their natural properties with nanotechnology for enhanced treatment outcomes.

Moreover, induced pluripotent stem cells (iPSCs), such as CAR-NK cells and CAR macrophages, are emerging as promising sources for generating CAR-expressing immune cells [249, 250]. iPSC-derived CAR-expressing macrophages (CAR-iMacs) exhibit robust antigen-dependent functions, including proinflammatory cytokine secretion, enhanced phagocytosis of tumor cells, and polarization toward an antitumor phenotype. By utilizing iPSCs as a renewable source, this approach could mitigate the high costs and complexities associated with the personalized manufacturing of CAR-T-cell therapies [249]. For CAR-expressing iPSC-derived NK cells, a CAR containing NKG2D-2B4-CD3ζ could mediate robust antitumor function. Mechanistic studies revealed the activation of NK-specific signaling pathways, promoting stable effector function and greater tumor control than T-cell-based CAR therapies [250]. In conclusion, the evolution of engineered cell therapies into multiple immune cell types represents a transformative approach to treating various diseases. The ability to harness and modify the immune system offers unprecedented opportunities to engage with challenging medical conditions.

## Summary

In this review, we sought to determine how synthetic CARs and TCR-like receptors leverage principles from native TCRs to further broaden T-cell functionality beyond tumor applications. We began by introducing native receptors, including TCR-CD3, costimulatory receptors and cytokine receptors, and their essential roles in T-cell activation and response, highlighting critical insights gleaned from natural immune mechanisms. Building on these foundational principles, we explored various synthetic receptors, focusing on CARs and TCR-like receptors that harness key attributes from their native counterparts to improve specificity, efficacy, and safety. In addition to synthetic receptors, cellular modifications, such as gene editing, have emerged as pivotal tools for refining synthetic receptor design and engineering more durable and versatile T-cell populations. Our discussion then expanded to engineered T-cell therapy in potential applications beyond tumors, recognizing the need to address diverse pathological contexts. Finally, we assessed emerging strategies involving combinations of multiple cell types, which hold promise for more robust and coordinated immune responses.

Despite these advancements, several obstacles remain. The potential for immunotoxicity and off-target effects underscores the importance of developing safer strategies, but their limited persistence and functionality in certain physiological environments continue to pose challenges. Future research should therefore focus on optimizing vector designs, refining gene-editing methodologies, and investigating how combination therapies can maximize therapeutic outcomes. By integrating insights from natural immunity with receptor engineering techniques, engineered T-cell therapies for cancer and autoimmune diseases ultimately advanced the field toward more effective immunotherapies.
